# Green synthesis and characterization of gold nanoparticles from *Pholiota adiposa* and their anticancer effects on hepatic carcinoma

**DOI:** 10.1080/10717544.2022.2056664

**Published:** 2022-04-01

**Authors:** Zhongwei Yang, Zijing Liu, Junmo Zhu, Jie Xu, Youwei Pu, Yixi Bao

**Affiliations:** a Department of Clinical Laboratory, The Second Affiliated Hospital of Chongqing Medical University, Chongqing, China; b Department of Gastroenterology, The Third Affiliated Hospital of Chongqing Medical University, Chongqing, China

**Keywords:** *Pholiota adiposa* polysaccharides, gold nanoparticles, immunoregulation, liver cancer

## Abstract

Gold nanoparticles (AuNPs) were successfully fabricated by *Pholiota adiposa* polysaccharide (PAP-1a) without employing any other chemicals. The physical and chemical properties of PAP-AuNPs were determined using transmission electron microscopy (TEM), dynamic light scattering (DLS), energy-dispersive X-ray spectroscopy (EDXR), Fourier-transform infrared spectroscopy (FT-IR), and atomic force microscopy (AFM). In an attempt to analyze the immune regulation, antitumor effect, and biological safety, the production of NO and TNF-α, IL-12p70, and IL-1β from RAW264.7 as well as the proliferation of RAW264.7 were detected *in vitro*. Flow cytometry was conducted to determine the ratio of the CD4^+^/CD8^+^ cell in peripheral blood and immunohistochemical analysis involving hematoxylin and eosin (H&E) and proliferating cell nuclear antigen (PCNA) staining were conducted *in vivo*. The results of this study showed that PAP-AuNPs had a significantly improved immune regulation and anti-tumor effect in comparison to PAP-1a alone. PAP-AuNPs showed no toxicity both *in vivo* and *in vitro*. This study demonstrates a useful application of PAP-AuNPs as a novel nanomedicine for hepatic carcinoma.

## 1. Introduction

Recently, there has been a substantial increase in the application of nanomaterials in medicine. Gold nanoparticles (AuNPs) optical, plasma resonance, and bioconjugation properties may facilitate enhanced stability and allow AuNPs to easily integrate with biological molecules (Liu et al., 2019). Therefore, AuNPs are being extensively applied to many fields within the biomedical industry (El-Sayed et al., 2005; Bagheri et al., 2018). Besides, AuNPs have also emerged as a useful tool in the field of diagnosis and cancer treatment (El-Sayed et al., 2005; Bagheri et al., 2018; Khan & Khan, 2018). Nevertheless, when physical or chemical methods were employed for the synthesis of nanoparticles, the resulting nanoparticles were demonstrated to possess low efficiency and toxic side effects. Thus, the development of newer methods to synthesize the nanoparticles with minimal toxic side effects is highly in demand.

Traditional Chinese medicines (TCM), well known for their prominent immune regulation effects and minimal side effects, are being applied extensively in the medical field. Researchers frequently purify polysaccharides, phenolic acids, flavonoids, esters, and coumarins from Traditional Chinese Medicines to determine their pharmacological effects, which range from immunological modulation to anti-infection (Yu et al., 2018; Teng et al., 2020). *Pholiota adiposa* (PA) is a widely used edible and medicinal mushroom in traditional Chinese medicine. PA possesses antimicrobial, anti-obesity, anti-hypertension, and hypolipidemic properties. These activities are attributed to the polysaccharides, ergosterol, and amino acids existing in PA (Ramachandran et al., 2014). A polysaccharide derived from PA functions as the primary physiologically active component. *Pholiota adiposa* polysaccharide (PAP-1a) was isolated and purified based on earlier research and it was demonstrated that PAP-1a can regulate the immunological response of macrophages *in vitro*. We synthesized AuNPs using PAP-1a and subsequently investigated their immune modulation and anticancer properties while preventing the formation of any compounds with hazardous side effects during the drug purification process.

According to the Global Cancer Statistics 2021, the prevalence of hepatic carcinoma has been more or less unchanged in men but has been steadily increasing (2% annually) in women, with the primary causes being obesity, HBV, and HCV (Siegel et al., 2021). Nowadays, the most prevalent treatment of Hepatic carcinoma comprises surgical resection; Nevertheless, a majority of patients are diagnosed too late to allow surgical resection (Forner et al., 2018). While Sorafenib has proven to be a useful treatment, the average survival rate is no more than 3 months (Keating, 2017). Transarterial chemoembolization (TACE) was considered as a valid therapy for the patients who had an advanced level, unresectable hepatic carcinoma (Lo et al., 2002). Recent studies have demonstrated the good effects of the use of immunotherapy, such as nivolumab and atezolizumab, etc. for the treatment of Hepatic carcinoma (Yau et al., 2019; Lee et al., 2020; Pinato et al., 2020; Yang et al., 2020). On the contrary, liver cancers are usually prone to recurrence and a high rate of distal metastasis makes the available therapy effective only to a limited extent. Therefore, finding a new therapy for the treatment of Hepatic carcinoma is rather urgent.

**Figure 1.**
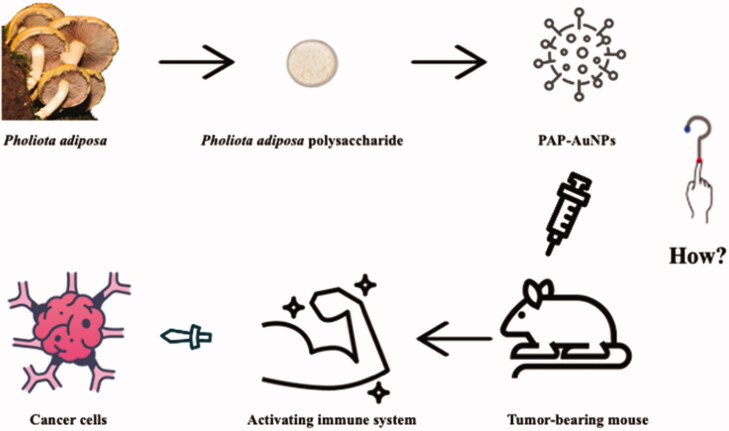
Conceptual diagram of PAP-AuNPs and its indirectly anti-cancer effect.

Numerous studies have demonstrated the green synthesis of AuNPs using TCM polysaccharides and extracts such as *Marsdenia tenacissima*, *Ulva intestinalis* L. aqueous, and Encapsulating Astragalus, among others, can inhibit tumor cell proliferation (Li et al., 2019; González-Ballesteros et al., 2019; Xiong et al., 2020). Because PAP-1a regulates the immune system and has indirect anticancer actions in liver cancer, AuNPs may enhance PAP-1a's biocompatibility. In this study, we employed PAP-1a to lower HAuCl_4_ and examined its influence on immune modulation and hepatocellular cancer, which may provide a novel therapeutic approach for hepatocellular carcinoma (Figure 1).

## 2. Methods and materials

### 2.1. Regents

*P. adiposa* was procured from a traditional herbalist. Prior to synthesizing PAP-AuNPs, we obtained the *P. adiposa* homogeneous polysaccharide (PAP-1a). Hyclone (Logan City, UT, USA) provided EDTA/trypsin and Dulbecco's modified Eagles medium (DMEM). Gibco Life Technologies (Grand Island, USA) provided fetal bovine serum (FBS). Adriamycin (ADM) was purchased from Main Luck Pharmacies Inc. (Shenzhen, China). Xiangding Biological Technology Co. Ltd (Shanghai, China) provided the chloroauric acid (HAuCl_4_) whereas G-CLONE Biological Technology Co. Ltd (Beijing, China) provided the trisodium citrate dehydrate. Boster Biological Technology Co. Ltd (Pleassanton, CA) provided the NO kits and cell counting kit-8 (CCK-8) test kit while the 4 A Biotech (Beijing, China) supplied the ELISA kits. Beckmen Coulter supplied CD3/CD4/CD8 antibodies (Navios, USA).

### 2.2. Synthesis of PAP-AuNP

3 mg/mL PAP-1a solution was prepared by dissolving 3 mg PAP-1a in 1 mL distilled water. Later, 1.5 μL chloroauric acid (1 mM) was added into the PAP-1a solution kept at 80 °C for 30 minutes (The optimization study of PAP-AuNPs was showed in Figures S1–S4). The change in the color of the liquor from light yellow to ruby red determined the successful synthesis of PAP-AuNPs. Several methods were subsequently employed to confirm the characterization of PAP-AuNPs.

### 2.3. Characterization of PAP-AuNPs

On account of their surface plasmon resonance, the synthesized gold nanoparticles, PAP-AuNPs were distinguished by the color change to a red wine color and their UV-visible spectrometer (UV–vis) maxima at 520–540 nm. Transmission electron microscopy (TEM) was used to determine the morphological characteristics, which included size and shape. Prior to TEM analysis, a drop of PAP-AuNPs aqueous suspension in water was deposited on a carbon-coated copper grid and allowed to air-dry. The functional groups of PAP-AuNPs were identified using the Fourier-transform infrared spectroscopy (FT-IR) spectra. Dynamic light scattering (DLS) was used to determine the particle size and zeta-potential of PAP-AuNPs. Furthermore, an atomic force microscope (AFM) was used to examine the surface morphology of PAP-AuNPs.

### 2.4. Cell culture

The RAW264.7 macrophages H22 cells were collected from Chongqing Medical University's Second Affiliated Hospital's Department of Hepatobiliary Surgery. The RAW264.7 and H22 cells were cultured in DMEM at 37 °C in a 5% CO_2_ atmosphere. DMEM was supplemented with 10% FBS and contained 100 μg/mL streptomycin and 100 units/mL penicillin.

### 2.5. No level

The NO content in RAW264.7 cell culture media was determined using the Griess technique. RAW264.7 macrophages were grown in a 24-well plate (5 × 10^4^ cells/well). To each well, varying amounts of PAP-1a and PAP-AuNPs (100–400 μg/mL) were added. After 24 hours of incubation, the cell culturing supernatant was collected and centrifuged for 20 minutes at 2000 rpm. Following that, according to the instructions, a NO detection kit was used to detect the NO level in each well. The absorbance of the sample at 540 nm was measured using a microplate reader.

### 2.6. Cytokines level

After 24 hours of treatment with DMEM, LPS (100 ng/mL), PAP-1a, or PAP-AuNPs (100–400 μg/mL), the culture supernatant was collected and TNF-a, IL-6, and IL-1 levels were determined using an enzyme-linked immunosorbent assay (ELISA).

### 2.7. Direct effect of PAP-AuNPs

The CCK-8 test was used to determine the viability of RAW264.7. In a 96-well plate (1 × 10^4^ cells/well), RAW267.4 cells were cultured. After cell adhesion, different concentrations of PAP-1a and PAP-AuNPs (100–500 g/mL) were applied to the well. The CCK-8 kit was used to determine the cell proliferation rate after 24 hours. The cell proliferation rate was calculated using the formula.

Cell proliferation rate (%)=([OD (experimental)−OD (Blank)])/([OD (control)−OD (blank)]) × 100%

### 2.8. Animal modeling

Female C57BL/6 mice (age: 6–8 weeks) were procured from the Animal Facility of Chongqing Medical University. After accommodating them for a week, we constructed a tumor-bearing mouse model by subcutaneously injecting tumor cells (H22) under the right axilla (4 × 10^5^ cells/mouse). After modeling, the mice were returned to their environment and fed standard food for one week before being randomly divided into four groups (*n* = 6 each). 30 mg/kg PAP-1a and PAP-AuNPs and 4 mg/kg Adriamycin were administrated to those mice by tail vein injection once 3 days for six times. Normal saline was used as the control.

Body weights and tumor sizes of the mice were recorded by using a weighing scale and a vernier caliper every two days: the following formula: (a*b^2^)/2 was used to count the tumor volume, with a and b respectively corresponding to the longest and shortest diameter of the tumor. Heart, liver, spleen, lung, kidney, thymus, and peripheral blood samples of mice were gathered after sacrificing them by neck dissection. The following formulas were used to count the organ index and tumor inhibition rate:

Organ index=spleen (thymus) weight/body weight×100%,

Tumor inhibition rate=(1−A/B)×100%

where *A* represents the weight of the tumors of the treated groups and *B* represents the weight of the tumors of the control group.

H&E and PCNA staining were performed on the 4% polyoxymethylene immersed tumor. The percentage of positive cells was calculated by randomly recording three horizons at *400 magnification.

The peripheral blood with anticoagulant was mixed with anti-CD3, PerCP-eFluor 710, anti-CD4, FITC, and anti-CD8a, PE antibodies. After incubating in the dark for 15 minutes, hemolysin was added and incubated again. Finally, after three PBS washes, the CD4^+^/CD8^+^ ratio was detected by flow cytometry (Beckman coulter, Navios, USA).

### 2.9. Data analysis

IBM SPSS Statistics 26.0 was employed to carry out statistical analysis. The results were analyzed using one-way ANOVA and expressed as the mean ± standard deviation. Statistical significance was defined as *p* < .05 or *p* < .01. All experiments have been conducted three times.

## 3. Results

### 3.1. Synthesis and characterization of PAP-AuNPs

#### 3.1.1. UV spectroscopy

**Figure 2.**
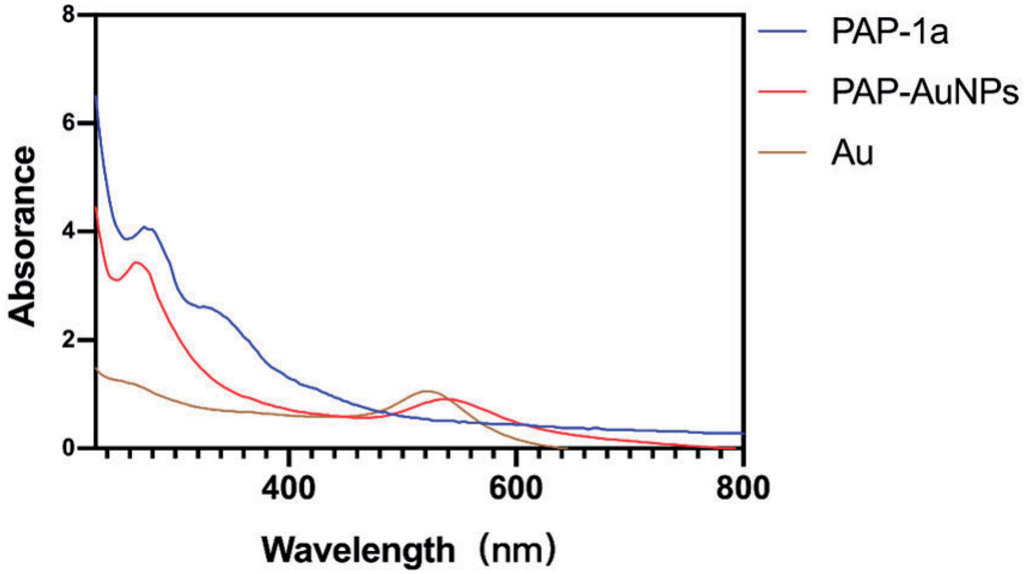
The UV–vis (240–800 nm) spectrum of PAP-1a, PAP-AuNPs and Au.

The green synthesis of nanoparticles has been a rapid development in various domains owing to their use in TCM (Markus et al., 2017). Whether or not the PAP-AuNPs were synthesized successfully was determined not only by the color variation (Venkatesan et al., 2014) but also by the ultraviolet–visible (UV) spectroscopy. There was an absorbance peak at 540 nm due to the surface plasmon resonance of AuNPs, as seen in Figure 2 which also corroborates well with the study of Rui (Liu et al., 2019) that states that the spherical shape nanoparticles give a strong absorption at 530 nm.

#### 3.1.2. Particle size detection

**Figure 3.**
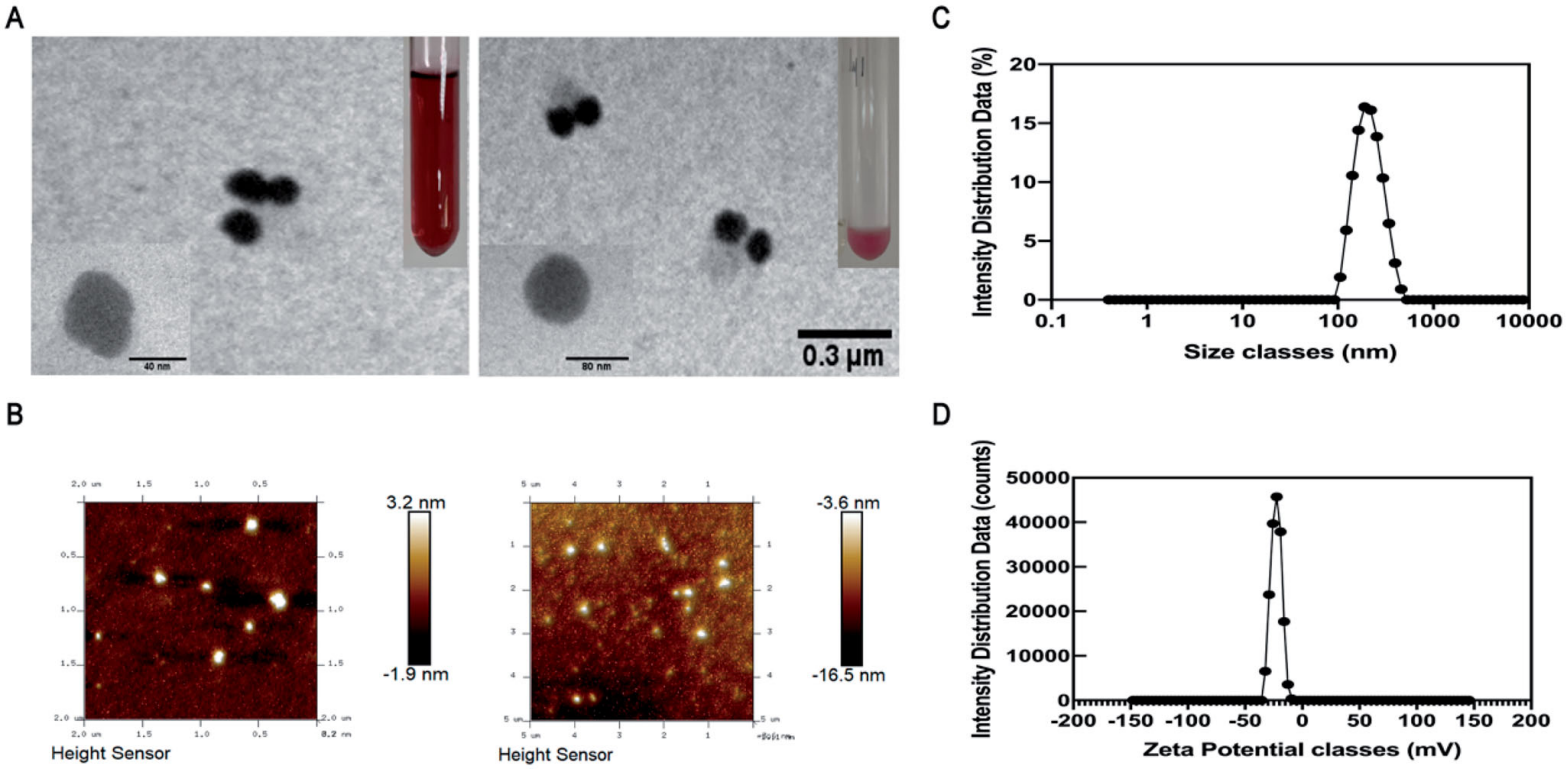
TEM image of PAP-AuNPs (A). (B) Atomic force microscopy analysis (AFM). (C, D) Dynamic light scattering (DLS) analysis.

The TEM picture of PAP-AuNPs (Figure 3(A)) revealed a spherical nanoparticle with a diameter of 70 nm, which was larger than Au nanoparticles alone (Figure 3(A)). And we also took TEM image of PAP-1a for contrast (Figure S5). Dynamic light scattering (DLS) was used to determine the size of PAP-AuNPs, which was estimated as 95.07 nm (Figure 3(C)). PAP-AuNPs have a good dispersion, as indicated by the PDI of 0.121. The DLS and TEM results were comparable. According to the results of TEM and DLS, there was a little uneven distribution of PAP-AuNPs, we thought that might be associated with the viscosity of PAP-1a as a kind of polysaccharides.

AFM was also used to examine the surface morphology of PAP-AuNPs (Figure 3(B,C)). The surface of PAP-AuNPs was rougher than that of Au nanoparticles which indicated that PAP-1a was successfully combined with Au. The AFM results were compatible with the TEM results, indicating that the PAP-AuNPs synthesis was successful.

*Pholiota adiposa* polysaccharide (PAP-1a) could stimulate the immune system and then suppress the formation of HCCs *in vitro*, according to preliminary research conducted by our research group. Because of the excellent drug delivery efficiency, PAP-AuNPs were able to greatly enhance the drug utilization of PAP-1a, and we anticipate that they can boost immunological regulation.

#### 3.1.3. Composition of PAP-AuNPs

**Figure 4.**
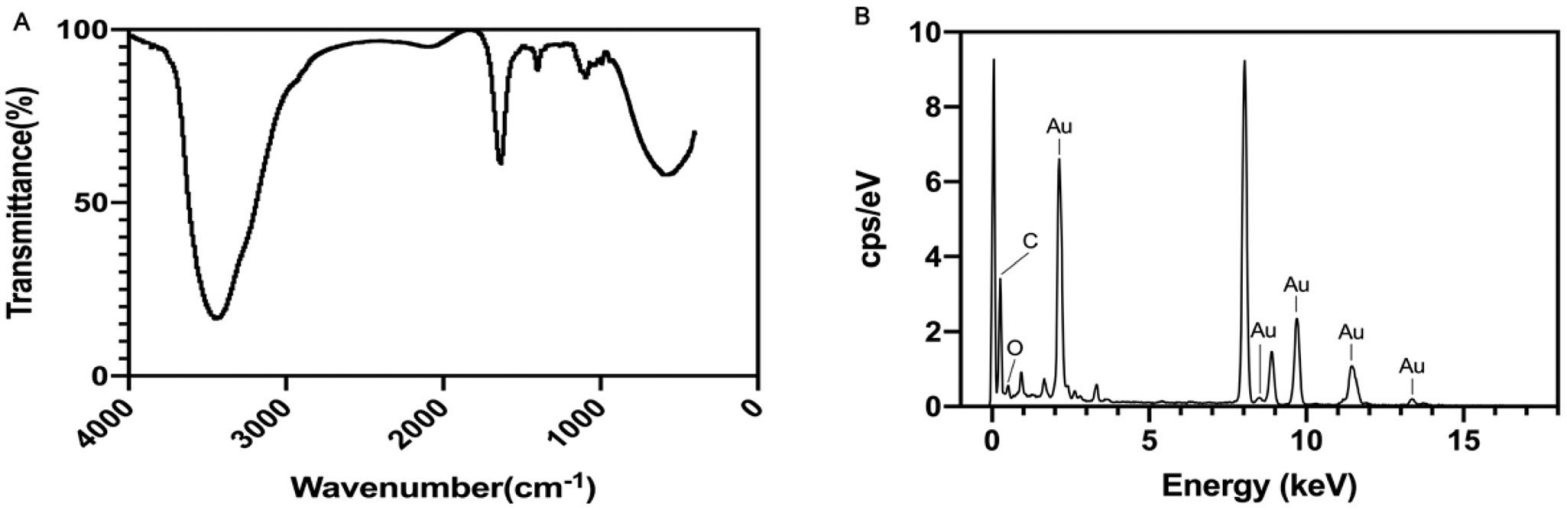
FT-IR (A) and EDAX (B) of PAP-AuNPs.

The FT-IR spectra of PAP-1a and its binding efficiency to AuNPs are shown in Figure 4. A. This result demonstrated the presence of multiple functional groups. The peaks observed for the PAP-AuNPs at 3443.09 and 1636.92 cm^−1^, which were correspondingly assigned to the free O-H and C=C stretching modes, could arise from the PAP-1a. Chemical, electrochemical, and photolytic reduction techniques are the most prevalent ways of the reduction of gold nanoparticles (Pestov et al., 2016). The reduction involved functional groups, such as hydro sulfonyl (–SH) and aldehyde group (–CHO) (Zhang et al., 2019). The FT-IR analysis of PAP-AuNPs revealed a peak at 1636.92 cm^−1^, indicating the presence of esters compounds and maybe revealing the underlying mechanisms of PAP-1a that bring about the reduction of chloroauric acid to PAP-AuNPs. The EDAX result depicted the elemental composition of PAP-AuNPs (Figure 4(B)) indicating the presence of Au, C, and O, which presumably aid in the effective synthesis of PAP-AuNPs.

#### 3.1.4. CCK-8 assay

**Figure 5.**
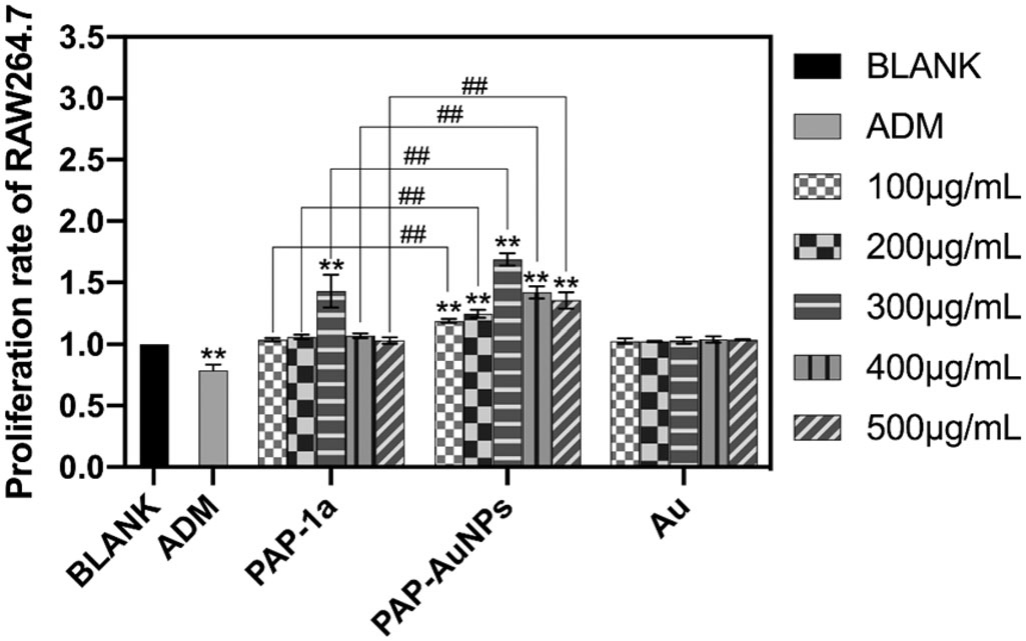
The biosafety of PAP-AuNPs *in vitro*. The proliferation rate of PAP-1a and PAP-AuNPs at various concentrations on RAW264.7. *p < .05, **p < .01. #*p* < .05, ##*p* < .01.

CCK-8 was used to assess the biosafety of PAP-AuNPs *in vitro*. PAP-1a (300 μg/mL) and PAP-AuNPs (100–500 μg/mL) significantly increased the viability of RAW264.7 cells after being cultured with varied concentrations of PAP-1a, PAP-AuNPs, and Au (100-500 μg/mL). PAP-AuNPs also showed better efficacy than PAP-1a. PAP-1a and PAP-AuNPs demonstrated good biocompatibility, as illustrated in Figure 5.

#### 3.1.5. The immune activate effect of PAP-1a and PAP-AuNPs

**Figure 6.**
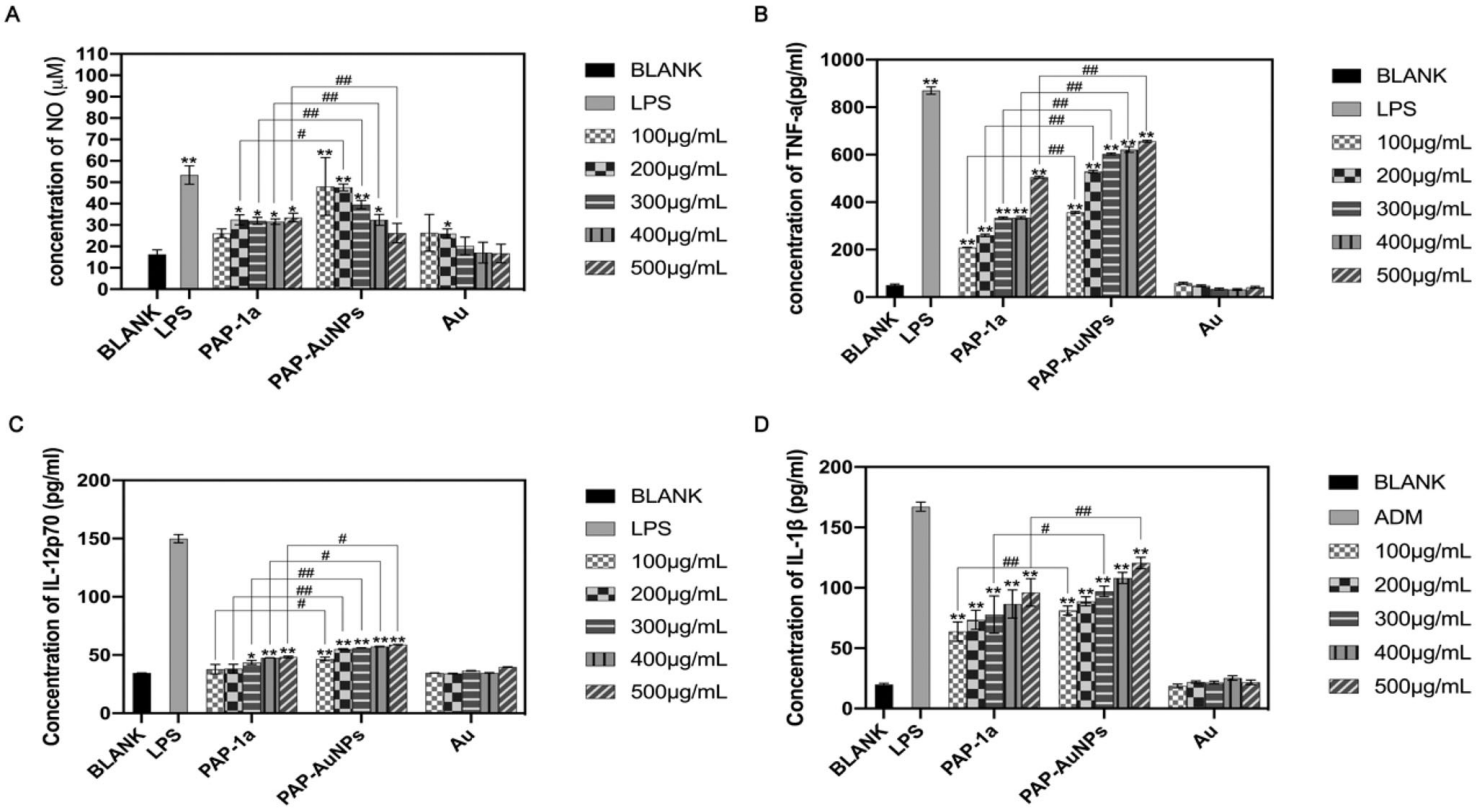
The stimulation effects of PAP-1a and PAP-AuNPs to RAW264.7 *in vitro*. The production of NO (A), TNF-α (B), IL-12p70 (C) and IL-1β (D). *p < .05, **p < .01. #*p* < .05, ##*p* < .01.

The production of NO changed progressively with the increase in the concentration of PAP-1a and PAP-AuNPs after 24-hour incubation with PAP-1a, PAP-AuNPs, and Au (Figure 6(A)). There was a significant difference in the generation of NO in the PAP-1a and PAP-AuNPs (100–500 μg/mL) exposed group in comparison to the blank control group. Furthermore, when exposed to PAP-AuNPs, there was a large increase in NO, which was similar in the case of the PAP-1a group. Au is unable to cause RAW264.7 to secrete NO. In the presence of PAP-1a and PAP-AuNPs, however, NO secretion was lower in comparison to that observed in the presence of LPS. These findings suggest that PAP-1a and PAP-AuNPs significantly activated RAW264.7.

To explore the effects of PAP-1a and PAP-AuNPs on the production of cytokines, ELISA was used to analyze the production of cytokines TNF-α, IL-12p70, and IL-1β. PAP-1a and PAP-AuNPs contributed to a dose-dependent increase in the cytokines. According to Figure 6(B–D), in comparison with the Normal saline (NS) group, PAP-1a and PAP-AuNPs could significantly promote the production of TNF-α (Figure 6(B)), IL-12p70 (Figure 6(C)) and IL-1β (Figure 6(D)). Compared with PAP-1a, PAP-AuNPs render a more important contribution in the stimulation of cytokines, but in comparison to the LPS group, PAP-1a and PAP-AuNPs could not trigger an inflammatory response, and Au had no stimulation effects on RAW264.7 cells. Based on these findings, we hypothesized that similar to PAP-1a, PAP-AuNPs may have an indirect anti-cancer effect via the production of NO and cytokines by macrophages activation.

### 3.2. Tumor inhibition *in vivo*

#### 3.2.1. Weight and volume

**Figure 7.**
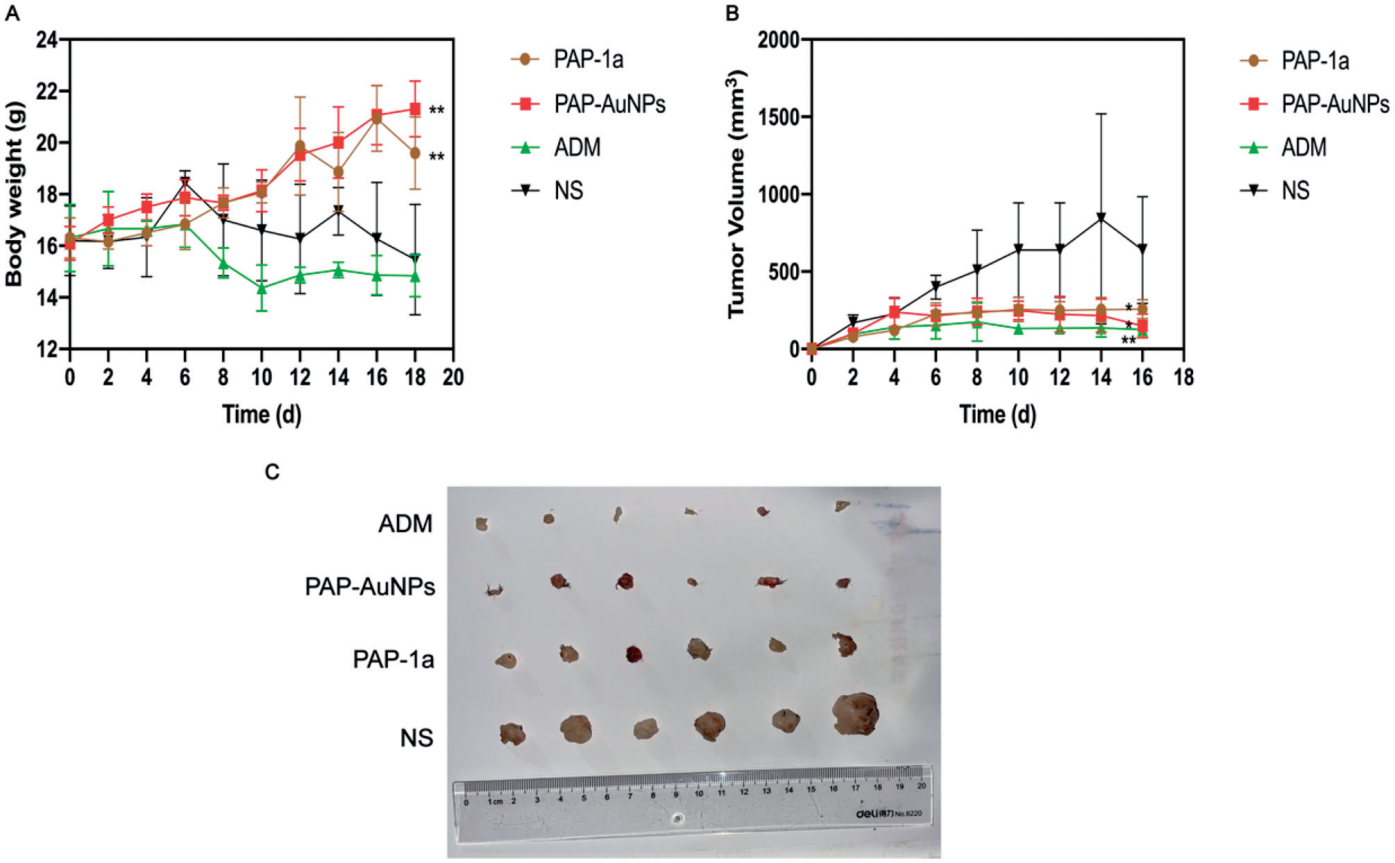
Gross tumor index. Body weight during the 20 days (A). Tumor volume curves of different groups during the 20 days (B). Photograph of tumors from different groups (C).

A traditional tumor-bearing mice model was established by using H22 (liver cancer cell) *in vivo*. As previously stated, the grouping is the same. PAP-1a and PAP-AuNPs demonstrated immunomodulatory and anticancer effects without noticeable side effects, as seen by steady weight changes in each group, as well as changes in body weight and tumor volume in mice. The fluctuation in body weight and tumor volume during the experiment is depicted in Figure 7(A,B). The tumors in the PAP-1a, PAP-AuNPs, and ADM groups grew slowly after 6 days, and their tumor weight steadily dropped, whereas others in the control group increased. Similarly, the photographs of the tumors (Figure 7(C)) the following sacrifice matched the prior results. PAP-1a and PAP-AuNPs have much better therapeutic benefits than the NS group. PAP-1A and PAP- AuNPs administered intravenously have therefore been found to have an anticancer effect (Figure 7(C)).

#### 3.2.2. Spleen index and thymus index

**Figure 8.**
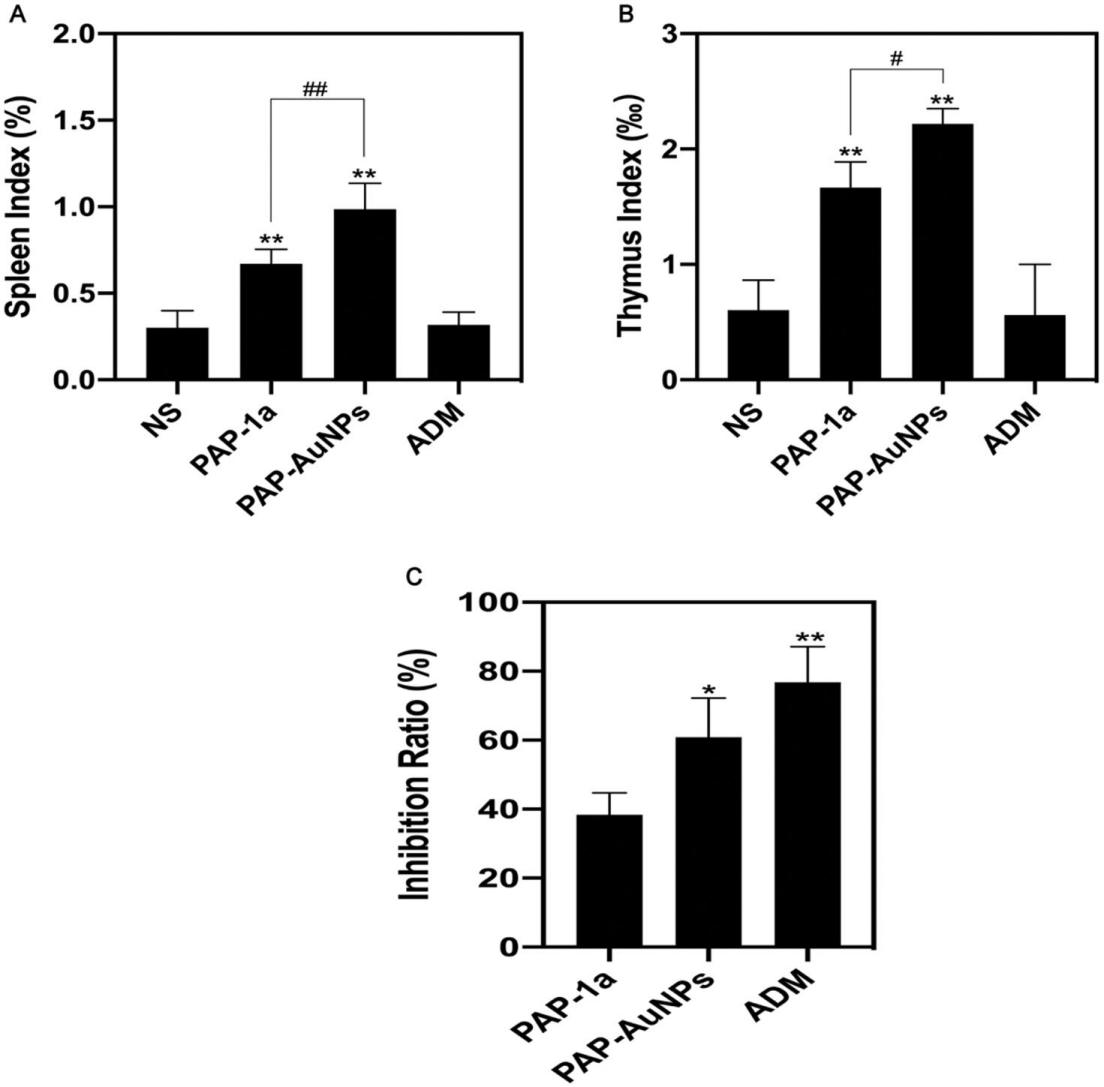
Tumor and organ index *in vivo*. Spleen and thymus index of different groups (A,B). Tumor Inhibition rate (C). *p < .05, **p < .01. #*p* < .05, ##*p* < .01.

Moreover, the spleen index and thymus index were found to increase in both PAP-1a and PAP-AuNPs groups and were relatively higher in the PAP-AuNPs group (Figure 8(A,B)). As suggested by our previous study, PAP-1a and PAP-AuNPs were able to stimulate the immune system *in vitro*. In addition, spleen and thymus indexes manifested an increase, and the PAP-AuNPs group’s immune regulation effects were significantly higher in comparison to the PAP-1a group *in vivo*. Thus, we anticipate that PAP-1a and PAP-AuNPs could also induce an indirect anticancer effect *in vivo*.

#### 3.2.3. CD4^+^/CD8^+^ T lymphocytes ratio

**Figure 9.**
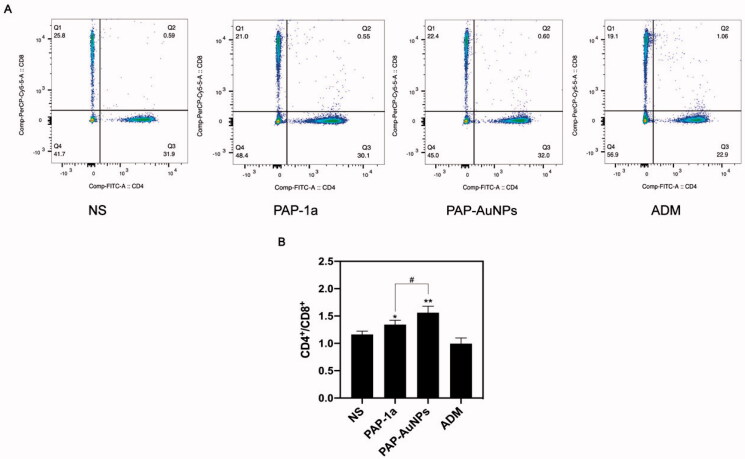
The CD4^+^/CD8^+^ ratio in peripheral blood. The results of flow cytometry (A). The CD4^+^/CD8^+^ ratio in peripheral blood in different groups. *p < .05, **p < .01. #*p* < .05, ##*p* < .01.

We measured the ratio of CD4^+^/CD8^+^ T cells in tumor-bearing mice to better understand their immune function. The CD4^+^/CD8^+^ ratio in peripheral blood was considerably greater in the PAP-1a and PAP-AuNPs groups than in the blank control group, as suggested by the findings (Figure 9). Figure 9(A) showed the results of flow cytometry of every group respectively, and Figure 9(B) showed the statistical results. PAP-AuNPs, on the other hand, have a better immune regulatory impact *in vivo*, whereas the ADM group showed no significant induction.

#### 3.2.4. Immunohistochemical analysis

**Figure 10.**
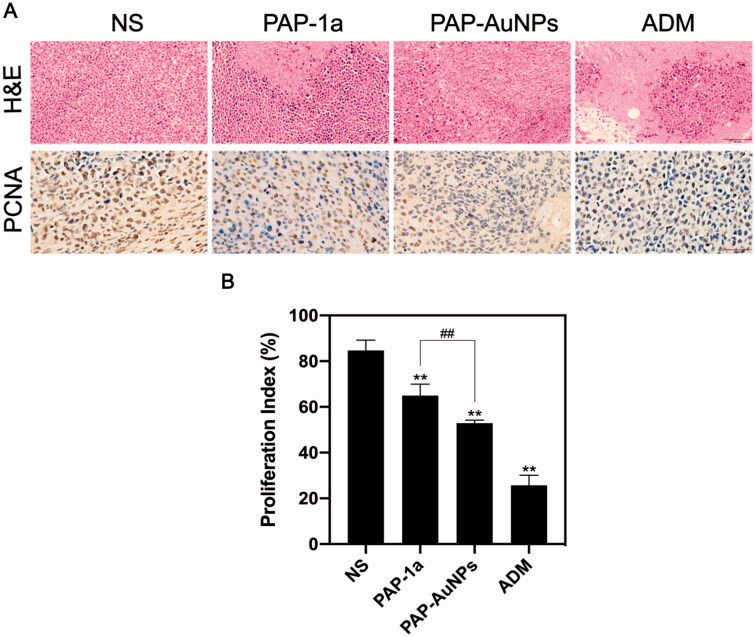
Histological index. H&E and PCNA staining of tumor in different groups (A). Proliferation index of different groups (B). *p < .05, **p < .01. #*p* < .05, ##*p* < .01.

To further test the effects of PAP-1a and PAP-AuNPs, H&E and PCNA were conducted on the tumors (Figure 10(A)). H&E staining revealed that the PAP-AuNPs group had a greater rate of necrosis. When comparing the PAP-1a and PAP-AuNPs groups to the NS groups, the number of PCNA-positive cells in the PAP-1a and PAP-AuNPs groups was considerably lower. Likewise, the proliferative index (Figure 10(B)) showed a similar tendency. These histological findings corroborate well with prior findings demonstrating the anti-cancer characteristics of PAP-AuNPs.

### 3.3. Toxicity of PAP-1a and PAP-AuNPs

**Figure 11.**
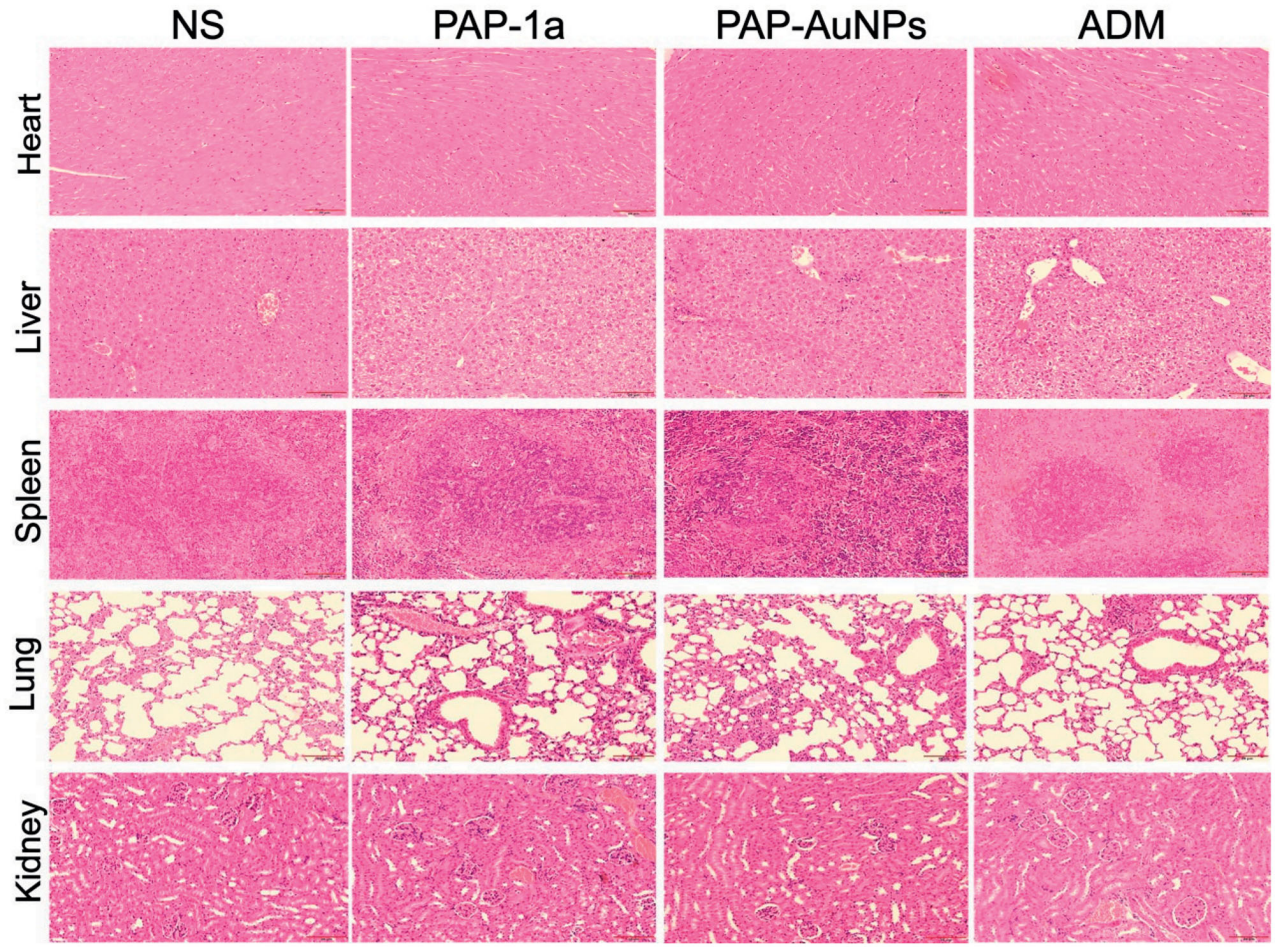
The toxicity of PAP-1a and PAP-AuNPs *in vivo*.

The toxicity of PAP-1a and PAP-AuNPs *in vivo* were detected by using the hepatic and renal function (ALT, AST, BUN, and sCr). These indexes indicated that PAP-1a and PAP-AuNPs can relieve liver and kidney injury to some extent, and the results of the biochemical analysis also revealed that ADM had significant liver and kidney damage (Table 1). Subsequently, H&E staining was conducted on the heart, liver, spleen, lung, and kidney. PAP-1a and PAP-AuNPs had no substantial toxicity or side effects in the tumor-bearing mice's vital organs. Although ADM had the best anti-cancer benefits, it also caused the most organ damage (Figure 11). These findings were in line with *in vitro* investigations that demonstrated the biosafety of PAP-1a and PAP-AuNPs. Overall, this research suggests that green synthesized nanoparticles could be a prospective treatment for liver cancer.

**Table 1.** The blood biochemical analysis of ALT, AST, Bun and sCr.

	ALT (U/L)	AST (U/L)	Bun (mmol/L)	sCr (μmol/L)
NS	50 ± 1.73	273 ± 46.81	10.27 ± 0.34	21.30 ± 0.26
PAP-1a	45 ± 1.00	194.33 ± 7.09**	9.74 ± 0.20	20.73 ± 0.06
PAP-AuNPs	42.33 ± 1.15*	161.33 ± 18.56**	8.54 ± 0.77**	19.30 ± 1.74
ADM	59.33 ± 5.69**	332.33 ± 10.12**	11.96 ± 0.31**	30.53 ± 6.24

*p < .05. **p < .01.

## 4. Conclusions

Nowadays, the use of Chinese herbal polysaccharide green reduction metal nanomaterials and synthesis of nano drug delivery system is more and more widely used. In this study, we successfully fabricated PAP-AuNPs from Traditional Chinese Medicine polysaccharide PAP-1a and demonstrated their immune activating function and indirect anti-cancer effect *in vitro* and *in vivo*. *In vitro*, PAP-AuNPs could significantly increase the production of NO and cytokines. Meanwhile, according to the results of CCK-8, PAP-AuNPs also increased the proliferation of macrophage cells *in vitro*. Then we estimate the indirectly anti-tumor potential of PAP-AuNPs *in vivo*. PAP-AuNPs could activate tumor-bearing mice’s immune system by increasing CD4^+^/CD8^+^ ratio in peripheral blood, spleen index and thymus index. Because of the immune activation effects of PAP-AuNPs, it showed significant antitumor effect *in vivo*. PAP-AuNPs could increase the tumor inhibition rate and H&E and PCNA staining also confirmed that inhibition effects. Overall, Traditional Chinese Medicine was employed for the reduction of HAuCl_4_, *via* a green technique with potential immune modulation and demonstrated anticancer effects *in vitro* and *in vivo*.

## Supplementary Material

Supplemental Material
