# Supplementary material for: Green synthesis and characterization of gold nanoparticles from Pholiota adiposa and their anticancer effects on hepatic carcinoma
Source: Drug Deliv. 2022 Apr 1;29(1):997–1006. doi: 10.1080/10717544.2022.2056664 (PMC8982465; doi:10.1080/10717544.2022.2056664)
Supplement: Supplemental Material [file IDRD_A_2056664_SM1368.docx]

**Supplymentary**

**
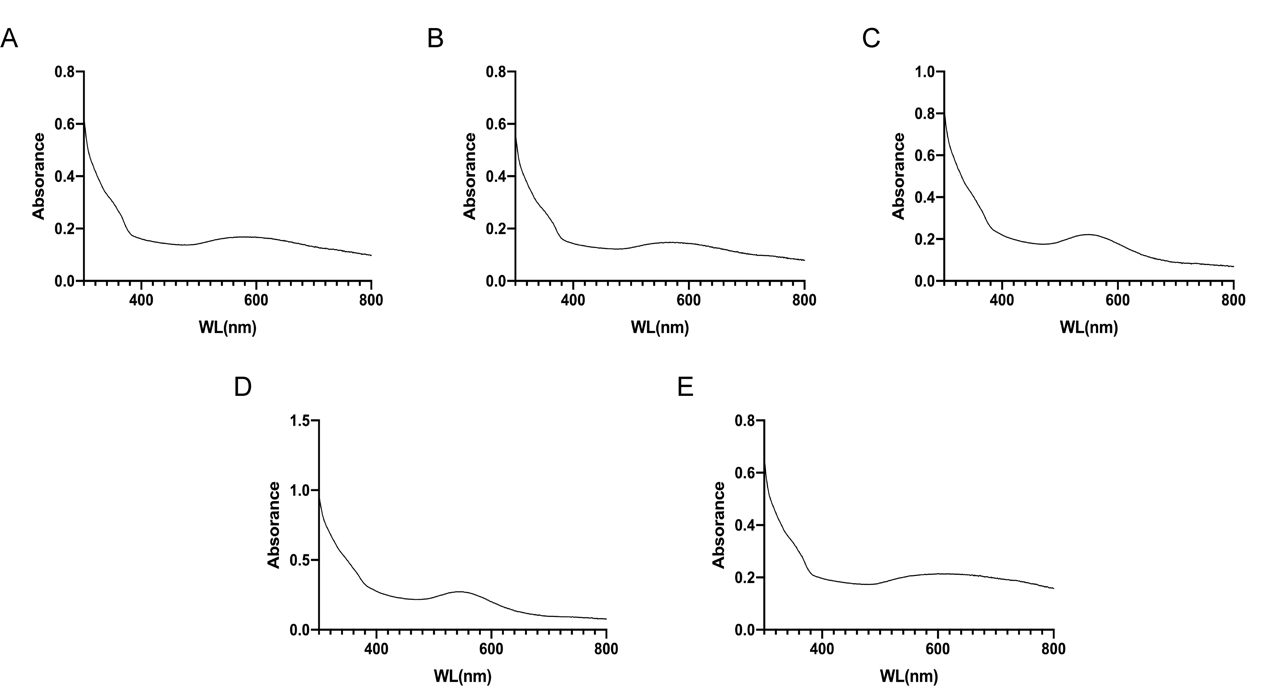
**

Figure S1. Volume of HAuCl_4_ (1M) optimization from 0.9 to 2.1 μl, 0.9 μl (A), 1.2 μl (B), 1.5 μl (C), 1.8 μl (D), 2.1 μl (E).

**
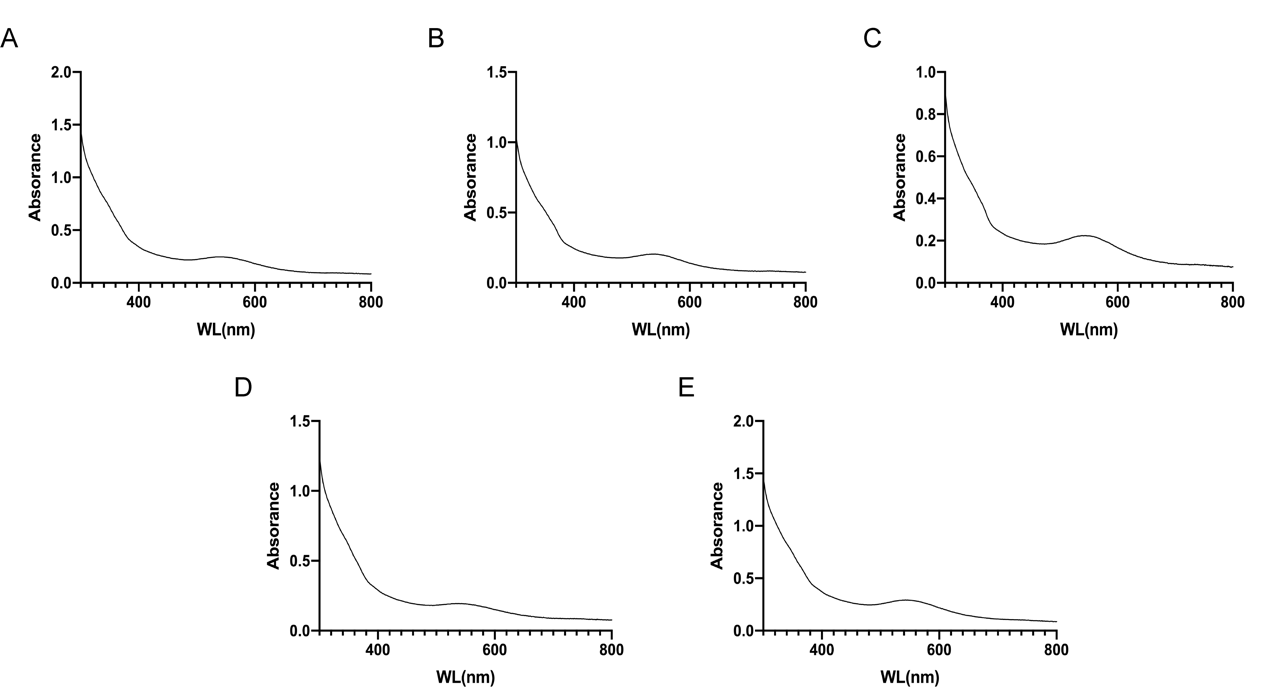
**

Figure S2. Concentration of PAP-1a optimization from 2.0 to 4.0 mg/mL, 2.0 mg/mL (A), 2.5 mg/mL (B), 3.0 mg/mL (C), 3.5 mg/mL (D), 4.5 mg/mL (E).

**
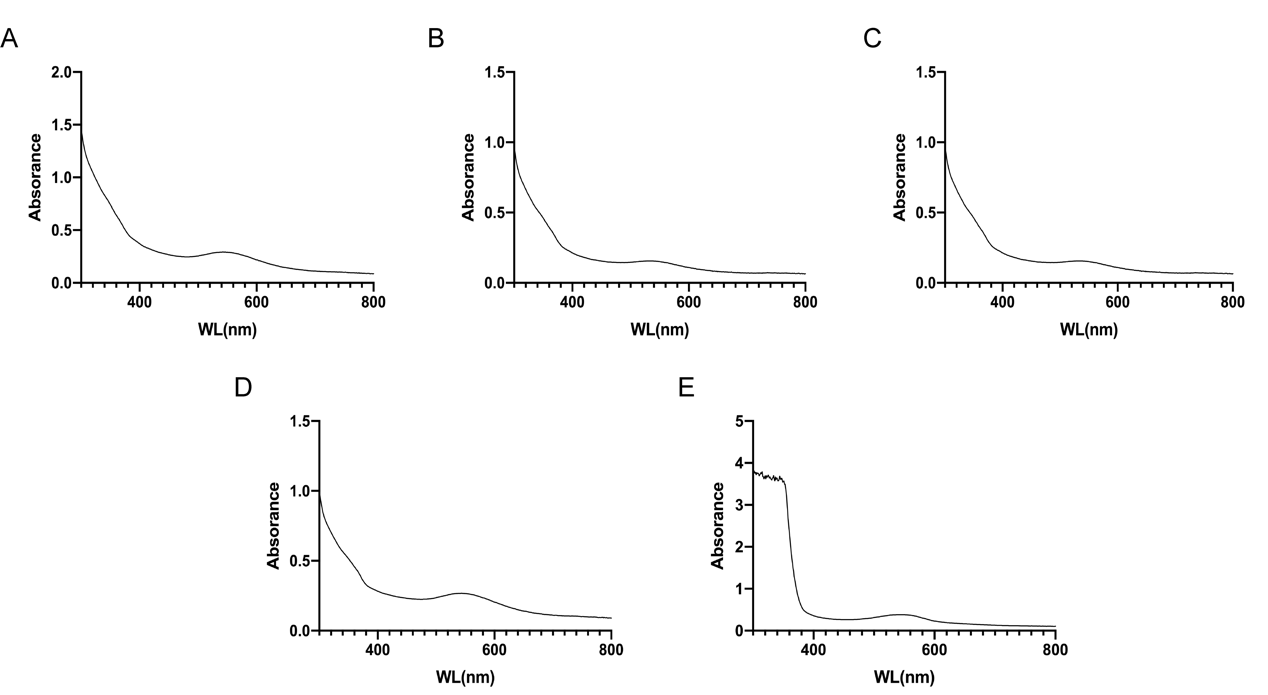
**

Figure S3. Temperature optimization from 60 to 100 C˚, 60 C˚ (A), 70 C˚ (B), 80 C˚ (C), 90 C˚ (D), 100 C˚ (E).

**
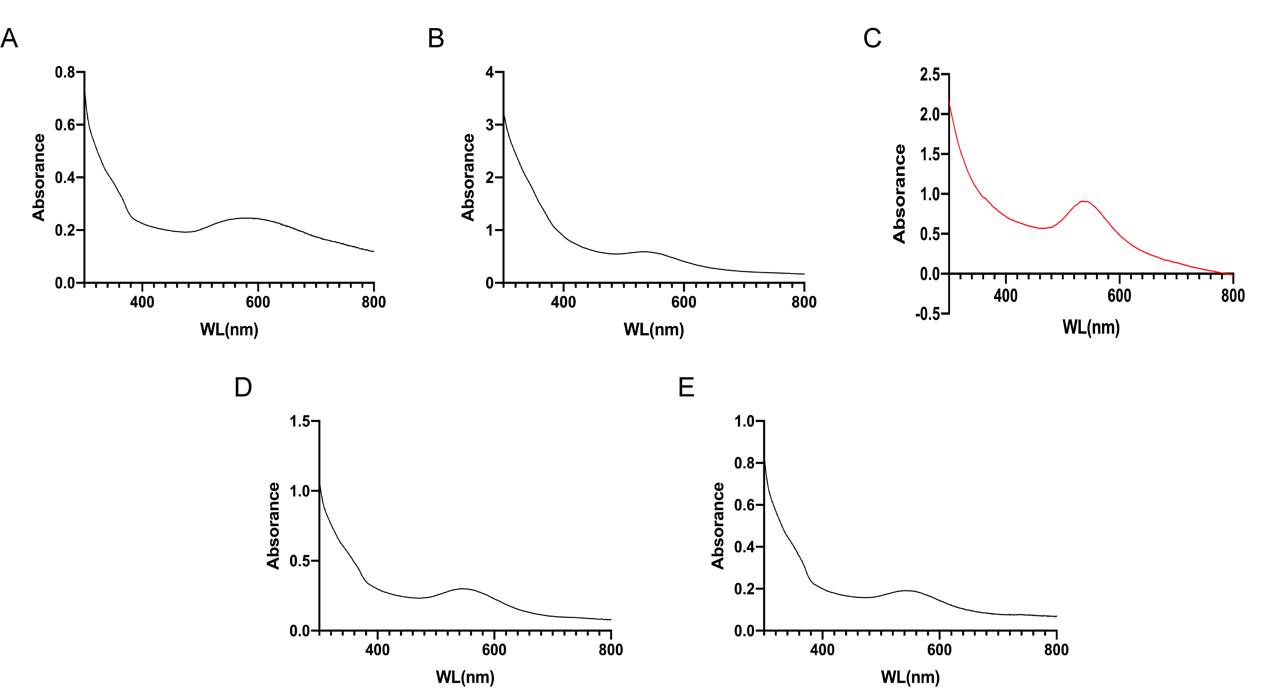
**

Figure S4. Time optimization from 20 to 40 minutes, 20 minutes (A), 25 minutes (B), 30 minutes (C, the optimal condition), 35 minutes (D), 40 minutes (E).


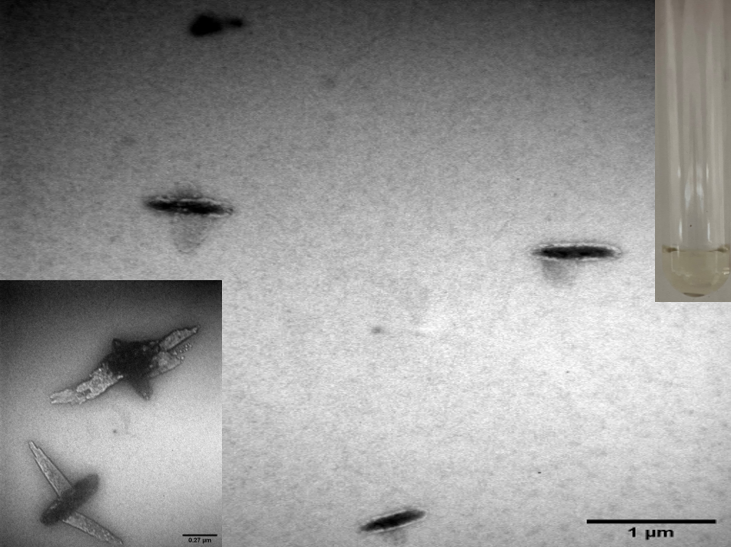


Figure S5. TEM image of PAP-1a.
